# Parametric and non-parametric Poisson regression for modelling of the arterial input function in positron emission tomography

**DOI:** 10.1186/s40658-023-00591-2

**Published:** 2023-11-21

**Authors:** Granville J. Matheson, Liner Ge, Mengyu Zhang, Bingyu Sun, Yuqi Tu, Francesca Zanderigo, Anton Forsberg Morèn, R. Todd Ogden

**Affiliations:** 1 Department of Biostatistics, Columbia University Mailman School of Public Health, New York, NY 10032 USA; 2 Department of Psychiatry, Columbia University, New York, NY 10032 USA; 3 Molecular Imaging and Neuropathology Division, New York State Psychiatric Institute, New York, NY 10032 USA; 4 Department of Clinical Neuroscience, Center for Psychiatry Research, Karolinska Institutet and Stockholm County Council, Stockholm, 171 76 Sweden; 5 Department of Biostatistics and Data Science, School of Public Health, University of Texas Health Science Center at Houston, Houston, TX 77030 USA; 6 Department of Neuroscience, University of Rochester Medical Center, Rochester, NY 14642 USA

**Keywords:** Positron emission tomography, Arterial input function, Principled statistical modelling

## Abstract

Full quantification of Positron Emission Tomography (PET) requires an arterial input function (AIF) for measurement of certain targets, or using particular radiotracers, or for the quantification of specific outcome measures. The AIF represents the measurement of radiotracer concentrations in the arterial blood plasma over the course of the PET examination. Measurement of the AIF is prone to error as it is a composite measure created from the combination of multiple measurements of different samples with different equipment, each of which can be sources of measurement error. Moreover, its measurement requires a high degree of temporal granularity for early time points, which necessitates a compromise between quality and quantity of recorded samples. For these reasons, it is often desirable to fit models to this data in order to improve its quality before using it for quantification of radiotracer binding in the tissue. The raw observations of radioactivity in arterial blood and plasma samples are derived from radioactive decay, which is measured as a number of recorded counts. Count data have several specific properties, including the fact that they cannot be negative as well as a particular mean-variance relationship. Poisson regression is the most principled modelling strategy for working with count data, as it both incorporates and exploits these properties. However, no previous studies to our knowledge have taken this approach, despite the advantages of greater efficiency and accuracy which result from using the appropriate distributional assumptions. Here, we implement a Poisson regression modelling approach for the AIF as proof-of-concept of its application. We applied both parametric and non-parametric models for the input function curve. We show that a negative binomial distribution is a more appropriate error distribution for handling overdispersion. Furthermore, we extend this approach to a hierarchical non-parametric model which is shown to be highly resilient to missing data. We thus demonstrate that Poisson regression is both feasible and effective when applied to AIF data, and propose that this is a promising strategy for modelling blood count data for PET in future.

## Introduction

Positron emission tomography (PET) is an in vivo imaging method for measurement of the concentration of specific proteins, peptides and biochemical functions. PET imaging involves the injection of a radiotracer into the blood which binds to a target protein in the tissue, while the emitted radioactivity over time allows its spatial origin to be measured using a PET system. The gold standard for the quantification of PET data is to measure the radioactivity in the tissue of interest as well as to measure the concentration of the parent compound in arterial blood plasma over time, and to fit a compartmental model to these data [18]. In this way, the measured arterial plasma samples, called the arterial input function (AIF) serves to describe the input to the tissue of interest. This is especially important for PET imaging of the brain, for which only the parent compound and not the radioactive metabolites, usually cross the blood-brain-barrier, necessitating the separation of plasma radioactivity into parent and metabolite fractions for accurate quantification. Using the AIF for PET quantification is referred to as *invasive* quantification as it requires arterial cannulation of participants, which is not only costly, but also uncomfortable. Moreover, these arterial measurements are prone to significant error, which can have implications for the fitting of the compartmental models [4, 16]. While there exist various methods for non-invasive quantification of PET data, such as the use of reference tissues, image-derived input functions, assessment of semi-quantitative outcome measures, among others [9, 39], all of these methods require additional assumptions which must be tested, and which cannot be justified for many applications. Invasive quantification of PET data, despite its challenges, will most likely remain an integral part of PET imaging for the foreseeable future [6].

The major difficulty with determination of the AIF is that it cannot be measured directly with the level of temporal granularity that we require to measure its rapid kinetics. However we can measure the radioactivity in arterial blood (whole blood, WB) in a large number of samples with relative ease, sometimes aided by the use of an automatic blood sampling system (ABSS), particularly at early stages of the PET examination, which can sample the blood radioactivity as quickly as every second. However, some of the whole blood radioactivity originates from radiotracer molecules which are bound to red blood cells and which therefore are not available to enter the tissue. For this reason, the blood must be centrifuged to measure the radioactivity concentration in the blood plasma. This is typically only performed in a subset of the whole blood samples. However, not all of the radioactivity in the blood plasma originates from the injected radiotracer compound itself: over the course of the PET examination, the radiotracer is metabolised by the liver and/or kidneys, resulting in radioactive metabolites in the blood. Using high-performance liquid chromatography (HPLC), we can separate the radioactivity in the blood plasma originating from the parent compound and from radioactive metabolites. However this is only usually performed in a subset of the arterial plasma measurements: usually between 3 and 10 per PET measurement. It is only by combining measurements and interpolating them over time that we can generate an AIF with the temporal granularity required for compartmental modelling of PET data. We typically do this by dividing the WB curve by the whole-blood-to-plasma ratio (BPR) curve to generate a whole plasma radioactivity curve. The whole plasma is then multiplied by the plasma parent fraction (PF) to estimate the metabolite-corrected arterial plasma radioactivity, or the AIF. The AIF must then be interpolated for the purposes of fitting the compartmental models. The interpolation of these functions is typically performed either using linear interpolation, or by fitting models to these data. Importantly, the resulting AIF is subject to the measurement error or fitting error from any one of these multiple curves, since the final AIF is made up of all of these measurements in combination with one another.

The way that blood data is collected, combined and interpolated differs not only between research groups, but also sometimes between analyses within research groups, in a fairly *ad hoc* manner. For instance, some groups make use of ABSS for measuring whole blood radioactivity (e.g. [5]), while others rely only on manual samples (e.g. [10]), or even forgo whole blood collection entirely, measuring only plasma radioactivity (e.g. [24, 28]). Different groups also apply various combinations of corrections to the measured samples. For instance, corrections for signal dispersion over time [17] can be applied for dispersion in ABSS tubing (external dispersion), as well as through blood vasculature (internal dispersion). Similarly, some groups correct for variation in the extraction efficiency of parent fraction measurements (e.g. [10]). After collection and correction of data, there also exist substantial differences in the approaches taken to interpolate these measurements. Models can be fit to the several of these curves, and some groups have historically been more or less skeptical of the use of models, and relying more or less on applying simple linear interpolation. The question of whether or not to make use of statistical models for this interpolation involves a bias-variance tradeoff. The use of a model results in smoother curves as estimates are informed by the whole set of measurements rather than fit to each individual data point independently, however it is possible that the applied model misses subtle true variation in the function. In this way, a model borrows strength across the whole series of samples recorded during the PET examination to better understand the value at each time point within the series. In our view, measurement error (i.e. variance) typically plays a more detrimental role in PET quantification than any potential underfitting (i.e. bias) that would be introduced by models fit to these data. Hence, we are broadly in favour of modelling these data, although this position may vary depending on the quality of the data, as well as the nature of the function being modelled. However, this position requires that caution should be exercised when applying models to these data in order to minimise the role of model bias.

For modelling of the AIF in particular, there exist several models in common use, including the sum of three exponentials (e.g. [11, 19, 28]), the Feng model [40] and its variants [37, 42], compartmental models [13], among others. What all of these models share in common is that they are fitted using nonlinear least squares, which implicitly makes the assumption of a Gaussian error distribution. One area in which this modelling can be improved is by taking the data-generating process into consideration and applying more principled statistical modelling and more appropriate error distributions by utilising generalised linear and nonlinear models [23]. More specifically, radioactivity concentrations are measured using a gamma counter, which measures the number of gamma photons emitted from the blood/plasma sample: this data is therefore *count* data. The error distribution most appropriate for modelling count data which are non-negative and never reach a theoretical maximum is the Poisson distribution, which has maximum entropy for data of this type [22]. Poisson regression is a type of generalised linear or nonlinear model which was developed over a half century ago, however, to our knowledge, no previous studies modelling AIF data have used this approach. By applying Poisson regression to count data, using either the Poisson or negative binomial distribution as its error distribution, optimal weighting is achieved *automatically* owing to the particular mean-variance relationships of these distributions. In contrast, models employing a Gaussian error distribution are forced to ignore the intrinsic mean/variance relationship that count data have, and instead devise an *ad hoc* weighting scheme to approximate the differences in error. While a Gaussian error distribution might work sufficiently well in some, or perhaps even most cases, it is not statistically principled for handling this type of data. While weighting strategies (e.g. [1, 37]) have been developed for use with Gaussian models, these are not only difficult to validate, but most importantly, owing to their unprincipled nature, they are a “Band-Aid solution” that does not address the source of the problem. In this way, by virtue of making use of a principled statistical modelling strategy, we gain greater efficiency and accuracy for fitting models to this type of data because the model automatically handles the error and weighting appropriately.

Here, we aimed to implement a Poisson regression modelling approach for application to AIF count data. Because of the *ad hoc* nature of blood modelling more generally, and the numerous factors and considerations which can impact the choice of modelling strategies, we position this study as a proof-of-concept to demonstrate the potential for application of statistically principled Poisson regression modelling of of blood radioactivity data. We evaluate relevant considerations for resolving issues encountered with the model specification, including using different error distributions, transformations of input variables, and the use of hierarchical models. We begin by demonstrating this approach using a simple parametric model for the AIF, and progressively developed the model through several evolutions to resolve encountered issues with the model specification.

## Materials and methods

### Terminology

During each PET measurement, there are a series of blood samples taken over time. For simplicity, *examination* will be henceforth used to describe the whole series of samples measured while a participant is in the PET system, while *sample* will be used to describe each individual blood sample recorded over the course of the examination.

### Model definition

#### Likelihood function

Poisson regression is commonly used for modelling variation in count data (i.e. 0, 1, 2,...) [12, 22]. It is a form of generalised linear and nonlinear models [23] which canonically uses the Poisson distribution as its data distribution. To build a generalised linear or nonlinear model, a link function is required. The log link is conventional for count data as it restricts the expected value (i.e. the rate parameter) to positive values, which is also important for the mean-variance relationships of these models as the variance cannot be negative. We have made use of the log link function and the following general model definition for all models presented below. The model is hence constructed as follows:

1 $$\begin{aligned}y_i \sim {\text {Poisson}}(\lambda _{i}) \quad \text{DATA DISTRIBUTION} \end{aligned}$$

2 $$\begin{aligned}\log {(\lambda _{i})} = f(t_i, \theta ) + \log {(\tau _{i})} \quad \text{NONLINEAR MODEL DEFINITION AND OFFSETS} \end{aligned}$$

From the data distribution, *i* represents each recorded sample during the PET examination, and $\lambda _i$ represents the corresponding Poisson rate, i.e. the expected value of the outcome $y_i$, which is an estimate of the number of counts. The log link is applied through the use of the log-transformation of the rate, $\log {(\lambda _{i})}$, in the model definition. $f(t_i, \theta )$ represents the model function used to describe the kinetics of the parent compound in the arterial plasma depending on unknown parameters, $\theta$, at time, $t_i$. Lastly, because $\lambda _i$ refers to the *rate* of events, it must take into consideration differences in exposure ($\tau _{i}$), e.g. if two blood samples are drawn at the same time, and sample A consists of twice as much blood as sample B, then we would expect approximately twice as many counts recorded for A—yet the rate of counts *per unit volume* is the same. For this reason, we incorporate these differences in exposure into the model through the use of the logarithm of the exposure, $\log {(\tau _{i})}$, which is called the model offset in the terminology of generalised linear and nonlinear models [12]. The model offsets are calculated a priori for each sample.

It should be noted that, in contrast to the Gaussian distribution which has mean $\mu$ and variance $\sigma ^2$ parameters, the Poisson distribution has only one parameter, the rate $\lambda$. This is because, for a Poisson random variable, the variance is exactly equal to the mean. However, this relationship is not always seen in practice for count data. In cases for which there is less or more relative variance in the number of measured counts, the data are said to be under—or over-dispersed, and the Poisson distribution is not able to accommodate this. This can be assessed from visual inspection of Q–Q plots [41], here generated using R [32] and the scam [30] and mgcv [44] packages.

For this reason, we also considered the negative-binomial (Eq. (3)) likelihood function, which is a useful generalisation of the Poisson distribution that can allow for over- or under-dispersion, i.e. when the variance is greater than or less than the Poisson model would indicate. When the negative binomial distribution was used, then we exchanged Eq. (3) for Eq. (1). Indeed, some texts claim that it is even *usually* appropriate in Poisson regression to include an additional parameter to capture overdispersion [12]. This use of the term *dispersion* is not to be confused with the external or internal dispersion of the measured radioactivity signal from its passage through tubing or vasculature respectively. To differentiate the two, we have always referred to statistical dispersion with over- or under-dispersion; and dispersion of the measured radioactivity signal as either internal or external dispersion. While the canonical parameters of the negative binomial distribution are the probability (*p*) and number of successes (*k*), these can be reparameterised to a dispersion parameterisation as an extension of the Poisson family as follows:

3 $$\begin{aligned} y_i \sim {\text {NegBin}}(\mu _{i}, \psi ) \end{aligned}$$

where $\mu$ represents the rate, analogous to $\lambda$ for the Poisson distribution, and $\psi$ is an additional parameter representing the dispersion such that the variance, equal to $\mu + \psi \mu ^2$, where $\psi =0$ corresponds to a Poisson distribution [2].

#### Nonlinear model

We made use of both parametric and non-parametric models. By parametric models, we mean that each of the parameters has a specific meaning in the context of the model, and that the predicted values can be generated from the model definition and the parameters themselves. By non-parametric models, we mean that parameters do not have any specific meaning in and of themselves, but only as a function of the basis functions generated [7, 14]. Hence, the generation of model predictions from nonparametric models requires the choice of basis functions as well as the corresponding estimated coefficients.

For the parametric model, we used a sum of three decreasing exponentials model as follows:

4 $$\begin{aligned} f(t_i, \theta ) = \sum _{k=1}^{3}{A_k e^{-\gamma _k t_i}} \end{aligned}$$

in which parameters $A_{1-3}$ and $\gamma _{1-3}$ must be estimated.

For non-parametric models, we made use of GAMs (generalised additive models) considering both general and shape-constrained basis functions. For shape-constrained functions, we used monotonically decreasing *B*-spline basis functions [31], and for basis functions without shape constraints, we used thin-plate basis functions [43]. Smoothing penalties were estimated using restricted maximum likelihood (REML), which has been shown to be less susceptible to undersmoothing compared to generalised cross-validation [33, 44, 45].

The choice of the basis dimension for smoothing terms is a modelling choice made as part of the model-building process when using penalised regression spline models [45]. In theory, it is not possible to select the basis dimension which is optimal, as this would require that we know the “true” smoothness of what is being estimated. In practice, setting the basis dimension sets an upper limit on the flexibility of a smoothing term, and the actual flexibility of that term is determined by the smoothing parameters. It is hence most important that the basis dimension is not set too low as to be excessively restrictive, but selection of a basis dimension which is not too high can also help to guard against overfitting without relying too heavily on suitable estimation of the smoothing parameters. A fuller discussion of these considerations, and the various diagnostics that can be used to guide appropriate selection, can be found in [45].

#### Offsets

In conventional estimation of the AIF, measured counts must first be converted to radioactivity concentrations, and then corrected in a series of steps finally to derive the arterial plasma parent radioactivity curve which constitutes the AIF, which can then be modelled. For the Poisson model, these correction factors are not applied directly to the measured data prior to modelling, but rather incorporated into the model itself as a series of offsets.

Importantly, the particular corrections and adjustments applied differ between research groups and data sets based on which data and parameters are recorded, the equipment used, and which are deemed necessary. As such, the particular corrections implemented here as offsets are not meant to represent an exhaustive list, and can be expanded or contracted depending on the particular dataset and application. For instance, we have performed external dispersion correction of ABSS samples (to correct for dispersion through tubing), but many groups consider this step unnecessary; and we have not performed internal dispersion correction (to correct for dispersion through vasculature) [17] or correction for extraction efficiency of the HPLC (e.g. [10]). The goal of this paper is to demonstrate the application of Poisson regression to AIF data as a proof of concept, and so we have made the decision to make use of the same corrections as the research group from which the data originated.

For all fitted models, we made use of the same combination of variables, described below in the rest of this section, to account for differences in exposure between samples. Offset variables were either directly measured, indirectly calculated, or estimated (see below). The total offset for each sample, $\log {(\tau _{i})}$, was defined as the sum of natural logarithms of all of the *M* offset variables, i.e.

$$\begin{aligned} \log {(\tau _{i})} = \log {(\tau _{1,i})} + \log {(\tau _{2,i})} + \cdots + \log {(\tau _{M,i})}. \end{aligned}$$

Directly measured offset variables include measurement duration in the gamma counter and sample volume. Indirectly calculated offset variables include radioactive decay relative to injection time, a volume calibration, and an external dispersion correction factor. Radioactive decay was calculated using the half-life of the radioisotope, in this case carbon-11. Volume calibration has historically been performed for the manual discrete data originating from this PET centre using a previously measured calibration function for the gamma counter to account for a very small deviation from linearity in its measured counts: $e^{\rho \cdot \text {vol}}$, where $\rho$ represents the calibration factor. External dispersion correction is applied for blood samples collected using the ABSS to correct for the dispersion over time of the measured signal owing to the sticking effect as the blood travels through the tubing. Here, dispersion correction was applied a priori to ABSS blood radioactivity values to estimate an external-dispersion-corrected blood radioactivity value for each ABSS sample using equations (5) and (6) from [25] implemented in kinfitr [21, 36]:

5 $$\begin{aligned} \int _0^t C_{\text{ true } }(T) \text {d} T =\kappa C_{\text{ meas } }(t)+\int _0^t C_{\text{ meas } }(T) \text {d} T \end{aligned}$$

where $\kappa$ represents the previously estimated dispersion constant of the system, and $C_{\text{ true } }$ and $C_{\text{ meas } }$ represent the true and measured blood radioactivity concentrations respectively. This equation is solved for $C_{\text{ true } }(t)$ by interpolating (5) over a uniform grid with spacing $\Delta t$ and estimating $C_{\text{ true } }(t)$ as follows:

6 $$\begin{aligned} C_{\text{ true } }(t)&=\frac{\int _0^{t+\Delta t} C_{\text{ true } }(T) \text {d} T-\int _0^{t-\Delta t} C_{\text{ true } }(T) \text {d} T}{2 \Delta t} \end{aligned}$$

For the purpose of incorporating external dispersion correction into the model as a multiplicative offset, we calculated a dispersion-correction factor by dividing the dispersion-corrected radioactivity values by the uncorrected values. For all manual samples collected after 10 min, the dispersion factor was set to 1, i.e. no correction, as these samples did not travel through the tubing.

Lastly, modelled offset variables included the BPR and the parent fraction. BPR is used to estimate the plasma radioactivity for samples in which only the whole-blood radioactivity was measured (such as the ABSS samples). For the BPR, using all manual samples for which both whole-blood and plasma radioactivity were measured, we fit a thin-plate regression spline with a basis dimension of 10 to the ratio of whole-blood to plasma radioactivity for each examination using mgcv [44]. For the offset, model estimates were estimated for all time points in which only whole-blood radioactivity was measured, and the BPR was set to 1 for all plasma samples. Similarly, for the plasma parent fraction, we fit a sigmoid function as described in [15], which has previously been validated for the modelling of [^11^C]PBR28 parent fraction data [26], to all parent fraction samples for each PET examination, and estimated parent fraction values for each time point.

### Subjects and data

As a case of study, we considered here the [^11^C]PBR28 data first reported by [5], which describes the full measurement and acquisition protocols. We used a subset of 10 individuals for whom the original whole blood and plasma count data were available. For each participant, arterial whole blood was sampled each second for 10 min using an ABSS (Allogg, Mariefred, Sweden). Background radioactivity, measured as the mean number of counts in the whole blood radioactivity measurements prior to the rapid ascent for each participant, was subtracted from these measurements by calculating the mean number of counts recorded per second in the samples recorded prior to injection, and subtracting this number from the counts recorded each second after injection. In parallel, manual arterial blood samples of 1–3 mL were drawn at 1, 3, 5, 7, 9, 10.5, 20, 30, 40, 50, 60, 70, 80 and 90 min post radiotracer injection. Manual samples collected before 10 min were drawn from the end of the tubing of the ABSS system, while samples recorded after 10 min were drawn directly from the arterial cannula without travelling through the tubing. Radioactivity was measured in each sample, followed by centrifugation to obtain between 0.8 and 1.5 mL of arterial plasma, in which radioactivity was also measured. Plasma parent fraction was measured at 1, 3, 5, 10.5, 20, 40, 60 and 90 min after injection.

We fit all Poisson regression models described below to the samples following the peak, i.e. the descent of the curve. This constitutes the majority of the measured curve, and the parametric tri-exponential AIF model is often applied to only this section of the curve with either linear interpolation (e.g. [11, 19]) or a linear model (e.g. [24, 28]) applied to the short ascending phase. It should be mentioned that there are are other parametric AIF models which can fit the whole curve though [37, 40, 42]. By focusing on the descending part of the AIF, we also avoid having to accommodate the discontinuity in our smooth spline nonparametric models, as the focus of this manuscript is on demonstrating the use of Poisson regression for AIF modelling.

### Software

All modelling was performed using R [32], using the gnm [38] package for parametric modelling, the scam [30] package for shape-constrained GAMs, and mgcv [44] for GAMs without shape constraints. All data and R code are provided in an open repository: https://github.com/77liner/ModelAIF.

## Results

### Parametric nonlinear Poisson regression

We started by applying a common parametric model, a sum of three decreasing exponentials (Eq. 4), using nonlinear Poisson regression. We derived starting parameters using the curve-stripping technique [20]. From visual inspection, this model exhibited a reasonably good fit to the data (Additional file 1: Supplementary Materials S1), although the model was prone to bias towards the end of the curve with predicted values lower than the final measured values for every fitted examination. This suggests that the tri-exponential model is likely underfitting the data, resulting in bias at later time points in this dataset. For this reason, we did not pursue this particular parametric model further.

### Non-parametric poisson regression

In order to define a more generalisable model with greater flexibility, we applied a non-parametric model. Because AIF data following the peak is expected to decrease throughout the descent, we applied a shape-constrained additive model with 15 knots, using a Poisson likelihood function. We observed a small degree of bias in the ABSS samples relative to the manual samples, in that the ABSS samples tended to be slightly higher than the manual samples over the same times. For this reason, we included an additional fixed effect describing an indicator term for whether data were collected using the ABSS system (as opposed to manually). This resulted in good fits to the data from visual inspection.

Despite the correspondence between the model predictions and the data, the residual Q–Q plots for the error distribution indicated clear overdispersion (Additional file 1: Supplementary Materials S2), implying that the Poisson likelihood function does not adequately describe the variance of the data. This means that the model will be excessively influenced by extreme values.

### Negative-binomial error distributions

In order to address the issue of overdispersion, we used a negative binomial likelihood function, i.e. exchanging Eq. (3) for Eq. (1).. This distribution is not available in the scam [30] package, so we used the mgcv [44] package instead. This package does not allow the use of shape constraints of the basis functions, so we instead applied an unconstrained thin-plate spline with a basis dimension of 15 [43]. With the same model definition otherwise, there was a tendency of the model to be excessively flexible, exhibiting clear non-monotonicity during the descent owing to the lack of shape constraints (Additional file 1: Supplementary Materials S3). This issue was not resolved either by increasing the smoothness penalty or by changing the number of basis functions.

**Fig. 1:**
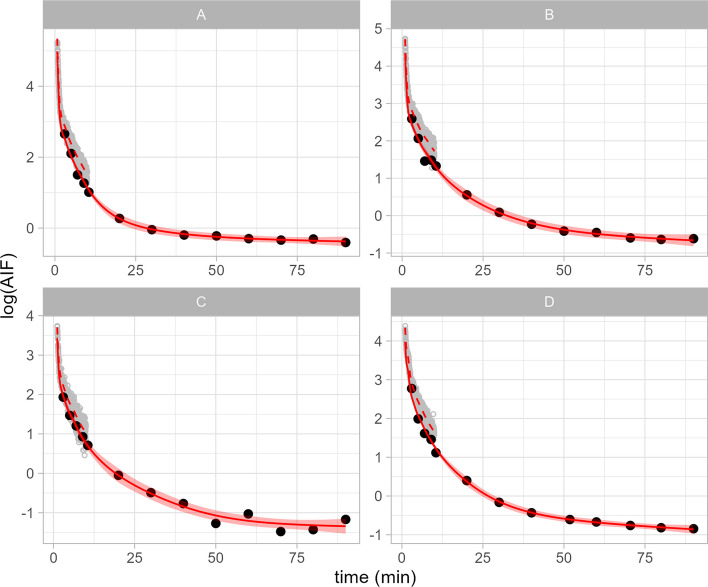
Non-parametric fits to the data for fit independently to data from four representative examinations. The large black points represent the manual discrete data, the small grey points represent the continuous automatic data. The solid red line represents the fitted curve, the shaded region represents the 95% credible interval around the fitted line, and the dashed red line represents the predictions for the continuous sampler after accounting for the estimated bias relative to the manual samples

We resolved this issue by log-transforming the time variable, i.e. $\log {(\lambda _{i})} = f( \log {(t_i), \theta }) + \log {(\tau _{i})}$. This results in a more even distribution of the collected data over time and, importantly, of the curvature of the function. In other words, the log-transformation results in expansion of the early time points with a higher second derivative and more samples, and contraction of the later time points with a lower second derivative and fewer samples. This serves to allow a more optimal spacing of the basis functions across the data, resulting in visually excellent fits without excessive flexibility (Fig. 1), and satisfactory Q–Q residual plots (all fits and Q–Q plots shown in Additional file 1: Supplementary Materials S4).

Examining difference in Akaike Information Criterion (AIC) scores for each fitted curve, as well as the mean difference in AIC scores, this was the preferred model. Compared to the model without the log-transformation of the time variable (mean $\Delta$AIC = $-222$, range: $-77$ to $-364$); compared to the non-parametric Poisson model (mean $\Delta$AIC = $-11167$, range: $-2302$ to $-26575$); and compared to the parametric Poisson model (mean $\Delta$AIC = $-5035$, range: $-935$ to $-12106$).

### Hierarchical non-parametric modelling

Owing to the lack of shape constraints and the ability of the negative-binomial error distribution to tolerate more extreme values than the Poisson, it is possible that such a model could overfit a curve owing to a small number of inaccurate samples. Hierarchical models are ideally suited to this issue as they borrow strength across individuals to yield better fits to each individual curve, providing an additional guard against overfitting. We hence applied a hierarchical GAM (HGAM) [29] to model data from all examinations at once. To this end, we defined fixed intercept differences for each of the 10 examinations, as well as for the interaction of the examination $\times$ ABSS indicator function to account for differences in the difference between manual and ABSS samples between examinations. For smooth functions, we defined a global mean smooth curve over the natural logarithm of time using a thin-plate spline basis with a basis dimension of 20, as well as a factor-smoother interaction term with thin-plate basis of dimension 5 for each examination. The latter are subject-specific smooth deviations away from the global mean smooth function which are penalised towards zero [29]. This resulted in good fits to the data and an acceptable residual Q-Q plot (Additional file 1: Supplementary Materials S5).

### Hierarchical modelling with incomplete data

A common problem in blood measurement is that of incomplete data owing to equipment malfunction or human errors. Through borrowing strength across all examinations, hierarchical models are especially helpful in these circumstances. To simulate an extreme example of incomplete data, we used a pseudo-leave-one-out approach, in which for each examination we modelled all of the data as if all but two manual samples from that particular examination were missing, while all the other 9 examinations had complete data. In this way, the HGAM model infers the shape of the mean AIF as well as the smoothness of the deviations from the remainder of the sample, and can thereby utilise the two non-missing samples from the incomplete examination most efficiently. We tested the model by leaving out all but the first and last samples, the first two samples, or the last two samples.

**Fig. 2:**
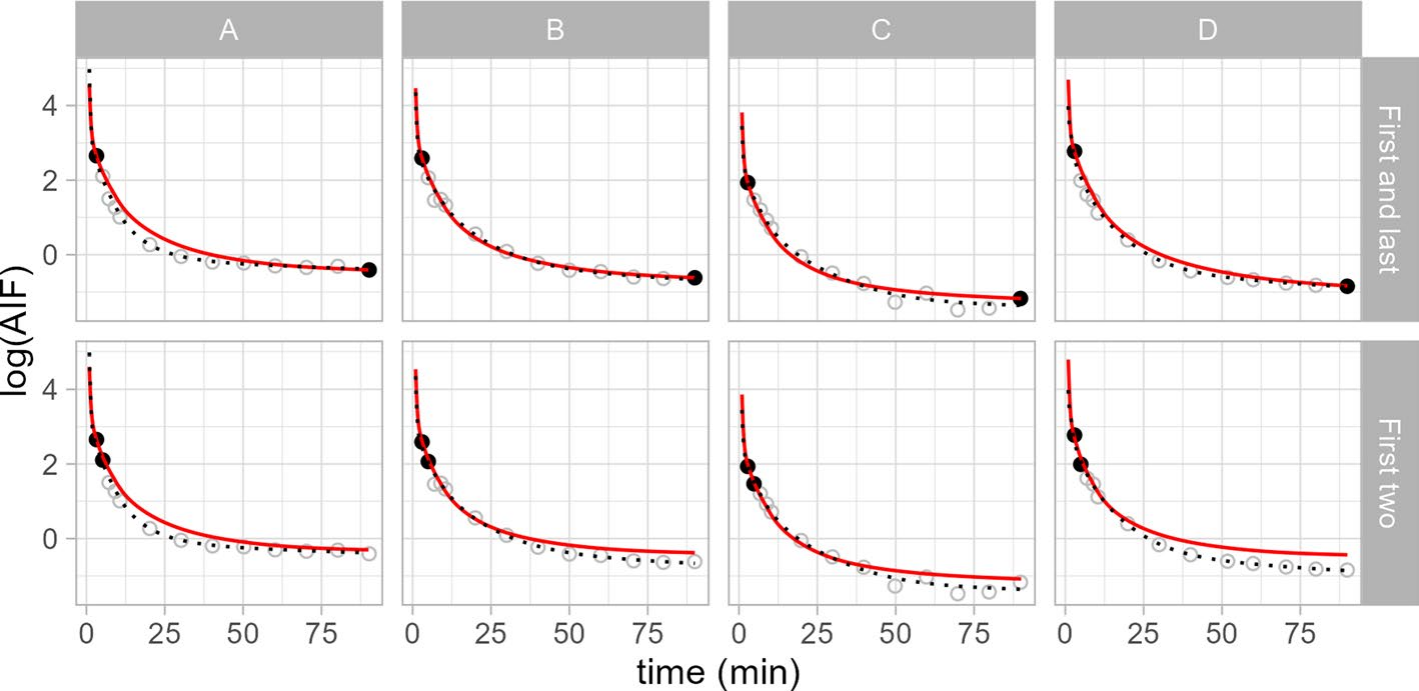
Hierarchical models leverage the rest of the sample to borrow strength, gaining resilience to missing data or outliers. The dotted black lines represent the negative binomial model shown in Fig. 1 fitted to all data points, while the red line represents the predictions of the hierarchical model fitted to the only two black data points from the specified examination alongside the rest of the sample: the first and last discrete data points (above), or the first two discrete data points (below) for four representative examinations. The grey points reflect the missing discrete data over which the model is extrapolating

In Fig. 2, we report the results for the same four examinations shown in Fig. 1, when either the first and last, or the first two blood samples are retained from the incomplete PET examination (all fits, as well as fits when only the last two samples are retained, are shown in Additional file 1: Supplementary Materials S6). In all cases, the model can infer the correct shape of the individual AIF function with reasonably high accuracy despite the extreme paucity of data from the specific examination itself.

## Discussion

In this study, we sought to model the AIF using Poisson regression applied to the raw count data in order to exploit the properties of the data-generating process and more accurately represent the error distribution. This *principled* approach ought to serve as a replacement for the use of nonlinear least squares with various *ad hoc* weighting schemes to fit these models. In this dataset, we show that Poisson regression can be successfully applied to model the data, that these models produce visually excellent fits to the data when applied at the individual level, with good residual distributions, and can also be extended to take account of similarities between examinations using a hierarchical modelling approach.

While the Poisson distribution provides the optimal likelihood function for count data under ideal conditions, this is not always true of real experimental data. We found that the Poisson distribution itself is insufficiently capable of describing the error distribution of the observed data owing to overdispersion. This additional variance in the recorded data may arise primarily as a result of experimental error, human error, or error in one or more of the offset variables, e.g. the modelled parent fraction curve. In fact, one previous study found that PET imaging data itself also showed overdispersion relative to the Poisson distribution [34]. Regardless of its cause, this additional variance can be effectively accounted for by the use of the negative binomial distribution which we found to be more consistent with the empirical error distribution.

Another issue of experimental data not corresponding with theoretical expectations is the mismatch between the radioactivity measured in manual samples and those from the ABSS. This could potentially have been caused by, for instance, the ABSS having been placed too close to the research participants. To this end, we incorporated a correction factor as an additional estimated parameter since we consider the manual samples to be the gold standard. This correction factor applies a uniform proportional difference between the manual and automatic samples for each PET measurement. Although it originally appeared as if the difference between measurement types was expanding with time, plotting the two curves with a log-transformed x-axis reveals that the difference is reasonably consistent over time, and that a proportional correction factor is justified (Additional file 1: Supplementary Materials S7).

For non-parametric models, we found that logarithmic scaling of time improved performance. This serves to more evenly spread out the rate of change in the slope of the rescaled function, since AIF curves typically descend much more rapidly following the peak and much less rapidly towards the end of the examination. For this reason, in practice we tend to collect more samples towards the start of the examination, and fewer towards the end. Although shape-constrained basis functions were able to still produce good fits to the data without this scaling, the fact that we could attain similar performance with a more flexible function is desirable for the modelling of other, more complex, AIFs such as for [^11^C]DASB, in which trapping and release of the tracer from the lung [27] can result in non-monotonic shapes after the peak.

We also demonstrated the use of hierarchical non-parametric models across multiple examinations [29]. To this end, we modelled a global mean curve, with regularised deviations from this function for each individual examination. These models therefore tend to be more robust owing to their ability to exploit similarities between individuals to describe the individual functions in a more constrained manner. Another advantage of these hierarchical models is that they can more effectively account for missing data by exploiting similarities between the measured curves. This is a common issue in PET examinations, as, for instance, blood samples could be physically dropped before measurement in the gamma counter, or a piece of equipment could fail during a period of the PET examination. We demonstrated the application of this using an extreme example in which only two samples are collected for one examination, showing reasonably good performance, especially considering for such a case, the model is simply not identifiable for either parametric nor non-parametric approaches applied at the individual level.

Because we require the time course of the AIF for pharmacokinetic modelling of PET data as opposed to estimating the specific underlying parameters, it is also possible to simply use the measurements themselves without fitting any model at all to the AIF data, or even to the series of measurements of which the AIF is comprised. Modelling of this data is therefore optional. A model might miss subtle, but true variation in the data, while the raw measurements themselves will include both the true variation, but also that which is caused by measurement error. Models fit to the data from each PET examination therefore borrow strength across the whole series of samples to improve estimation at each time point. However, even models can mistake measurement error for true variation, although to a lesser extent than the raw values. Hierarchical models are defined in such a way that commonalities between measured curves are identified by the model, and so these models should theoretically be better able to separate true variance from that cause by measurement error. In this way, these models can borrow strength not only between samples within examinations, but also between different examinations. However, this implies that if significant fluctuations in tracer availability at the individual level which are unique to that individual are expected, then hierarchical models might not be the best choice. Similarly, if significant true fluctuations in tracer availability at the second-to-second level are expected within measurements, which are important to be preserved, then fitting a model to this data at all is perhaps not to be recommended. However, we would propose that the application of principled models generally, and hierarchical models when possible, is desirable in most circumstances.

Our particular implementation of this model is limited in several respects. By accounting for modelled quantities such as parent fraction and BPR using offsets, we make the assumption within our model that these quantities are known, when in reality they are estimates. A more appropriate, but correspondingly more complex model would jointly fit all of these quantities, allowing the error in each to be propagated to one another. Similarly, accounting for external, or both external and internal, dispersion correction as an offset is also less than ideal: a better, though more complex, strategy would apply dispersion of the modelled “true” curve within the model to match the measured data, rather than an a priori dispersion correction of the measured data.

The model as demonstrated in this paper serves as a proof-of-concept for the application of Poisson regression to AIF data, and an exposition of some of the challenges encountered with this strategy and how they were overcome. The extent to which this approach improves modelling performance will primarily have to do with different sampling protocols and the amount of noise in any given dataset, but also which models are actually applied, as well as the tracer to which they are applied. In other words, this approach might provide only negligible benefits in some datasets, but large improvements in others. For instance, in this study there were continuous blood samples recorded in the first 10 min followed by manual samples collected throughout the remainder of the examination. This is only one of many potential sampling protocols which are in common practice in different PET centres: manual-throughout or continuous-throughout sampling protocols are also common alternatives. The collection of only manual samples would have resulted in a more temporally sparse sampling of the AIF but with higher accuracy of each individual point: this makes greater demands of a model’s ability to interpolate the appropriate function. In contrast, collection of only continuous samples throughout the examination would have resulted in a more temporally dense sampling of the AIF but with less accurate individual measurements, particularly after external dispersion correction: this therefore places greater demands on a model’s ability to handle noisier input data. Equipment may also be an important consideration for the quality of collected data as recently shown in [35]; as well as the number of staff available, and their individual level of experience and expertise for making these recordings, particularly in an academic setting; as well as a function of the radioligand being examined. Blood data will therefore differ between studies both within and between research centres, as a function of a host of factors. Although we would recommend principled statistical modelling generally as these models are more efficient, and should be more robust to extreme values, it would also be valuable for future studies to investigate the data properties which benefit most from the application of a principled modelling strategy.

In this paper, we focused on modelling the AIF descent following the peak. While there do exist parametric models which include both the ascent and descent (e.g. [8]), this can present some issues for non-parametric methods making use of smooth splines, since there is a very rapid change in the first derivative at the peak. While there are ways to handle this through either data transformations or careful knot placement, the performance of various approaches for handling this discontinuity should be evaluated in future studies. With regard to applying parametric models using this approach, we found that the negative binomial error distribution was most appropriate for this type of data. However, while this functionality exists for generalised linear models, there are to our knowledge no existing packages within the R [32] ecosystem which provide support for the negative binomial distribution for generalised nonlinear models. While this could be achieved using Bayesian modelling using STAN [3] for example, there is a need for a frequentist implementation of this approach.

PET and SPECT imaging are both fundamentally based upon measurement of radioactivity, which is originally recorded as counts. Here, we presented Poisson regression for application to modelling AIF data, however this is by no means the only application within the field where this approach might be applied, and there are a host of other potential applications for a principled modelling strategy. For instance, Poisson regression might be applied for the pharmacokinetic modelling of time-activity curves derived from the PET system directly. Another promising potential application of Poisson regression is for modelling parent and metabolite counts in HPLC data for analysis of radiotracer metabolism, especially considering the sparsity of the sampling of the parent fraction as well as the amount of noise towards the end of the measurement.

## Conclusions

In conclusion, by modelling count data using the appropriate modelling techniques, there is no need to devise *ad hoc* weighting schemes for models applying assumptions of normality of residuals. We show how we applied both parametric and non-parametric models, and how these could even be extended to hierarchical non-parametric models which are highly resilient in the presence of missing data. We propose that this is a promising modelling strategy going forward for modelling AIF data.

### Supplementary information

**Additional file 1.** Supplementary Materials.

## Data Availability

The code and data used to apply this method are provided in an open repository (https://github.com/77liner/ModelAIF).

## References

[1] Bassingthwaighte J, Chan I, Goldstein A (1988). An efficient method for smoothing indicator-dilution and other unimodal curves. Comput Biomed Res.

[2] Betancourt M. Probabilistic building blocks (2019). URL Retrieved from https://betanalpha.github.io/assets/case_studies/probability_densities.html

[3] Carpenter B, Gelman A, Hoffman MD (2017). Stan: A probabilistic programming language. J Stat Softw.

[4] Chen K, Huang SC, Yu DC (1991). The effects of measurement errors in the plasma radioactivity curve on parameter estimation in positron emission tomography. Phys Med Biol.

[5] Collste K, Forsberg A, Varrone A (2016). Test-retest reproducibility of [^11^C]PBR28 binding to TSPO in healthy control subjects. Eur J Nucl Med Mol Imaging.

[6] Correia J (1992). A bloody future for clinical PET?. J Nucl Med Off Publ Soc Nucl Med.

[7] De Boor C (1978). A practical guide to splines.

[8] Feng D, Wong KP, Wu CM (1997). A technique for extracting physiological parameters and the required input function simultaneously from PET image measurements: theory and simulation study. IEEE Trans Inf Technol Biomed Publ IEEE Eng Med Biol Soc.

[9] Feng DD, Chen K, Wen L (2020). Noninvasive input function acquisition and simultaneous estimations with physiological parameters for pet quantification: a brief review. IEEE Trans Radiat Plasma Med Sci.

[10] Finnema SJ, Nabulsi NB, Mercier J (2018). Kinetic evaluation and test-retest reproducibility of [^11^C]UCB-J, a novel radioligand for positron emission tomography imaging of synaptic vesicle glycoprotein 2A in humans. J Cereb Blood Flow Metab.

[11] Fujita M, Imaizumi M, Zoghbi SS (2008). Kinetic analysis in healthy humans of a novel positron emission tomography radioligand to image the peripheral benzodiazepine receptor, a potential biomarker for inflammation. NeuroImage.

[12] Gelman A, Hill J (2007). Data analysis using regression and multilevel.

[13] Graham MM (1997). Physiologic smoothing of blood time-activity curves for PET data analysis. J Nucl Med Off Publ Soc Nucl Med.

[14] Green PJ, Silverman BW (1993). Nonparametric regression and generalized linear models: a roughness penalty approach.

[15] Guo Q, Colasanti A, Owen DR (2013). Quantification of the specific translocator protein signal of 18F-PBR111 in healthy humans: A genetic polymorphism effect on in vivo binding. J Nucl Med.

[16] Huesman RH, Mazoyer BM (1987). Kinetic data analysis with a noisy input function. Phys Med Biol.

[17] Iida H, Kanno I, Miura S (1986). Error analysis of a quantitative cerebral blood flow measurement using H_215_O autoradiography and positron emission tomography, with respect to the dispersion of the input function. J Cereb Blood Flow Metab..

[18] Innis RB, Cunningham VJ, Delforge J (2007). Consensus nomenclature for in vivo imaging of reversibly binding radioligands. J Cereb Blood Flow Metab.

[19] Kim MJ, Lee JH, Juarez Anaya F (2020). First-in-human evaluation of [^11^C]PS13, a novel PET radioligand, to quantify cyclooxygenase-1 in the brain. Eur J Nucl Med Mol Imaging.

[20] Kirkup L, Sutherland J (1988). Curve stripping and nonlinear fitting of polyexponential functions to data using a microcomputer. Comput Phys.

[21] Matheson GJ (2019). kinfitr: reproducible PET pharmacokinetic modelling in R. preprint. Bioinformatics.

[22] McElreath R (2016). Statistical rethinking: a Bayesian course with examples in R and STAN.

[23] Nelder JA, Wedderburn RWM (1972). Generalized linear models. R Stat Soc J Ser A General.

[24] Ogden RT, Zanderigo F, Choy S (2010). Simultaneous estimation of input functions: an empirical study. J Cereb Blood Flow Metab.

[25] Oikonen V. Dispersion of input function (2019). http://www.turkupetcentre.net/petanalysis/input_dispersion.html

[26] Owen DR, Guo Q, Kalk NJ (2014). Determination of [^11^C]PBR28 binding potential in vivo: a first human TSPO blocking study. J Cereb Blood Flow Metab.

[27] Parsey RV, Ojha A, Ogden RT (2006). Metabolite considerations in the in vivo quantification of serotonin transporters using ^11^C-DASB and PET in humans. J Nucl Med.

[28] Parsey RV, Ogden RT, Miller JM (2010). Higher serotonin 1A binding in a second major depression cohort: modeling and reference region considerations. Biol Psychiatry.

[29] Pedersen EJ, Miller DL, Simpson GL (2019). Hierarchical generalized additive models in ecology: an introduction with mgcv. PeerJ.

[30] Pya N. scam: Shape constrained additive models (2021). https://CRAN.R-project.org/package=scam, R package version 1.2-12

[31] Pya N, Wood SN (2015). Shape constrained additive models. Stat Comput.

[32] R Core Team. R: A language and environment for statistical computing (2022). https://www.r-project.org/, tex. address: Vienna, Austria tex. institution: R Foundation for Statistical Computing

[33] Reiss PT, ToddOgden R (2009). Smoothing parameter selection for a class of semiparametric linear models. J R Stat Soc Ser B Stat Methodol.

[34] Rowe RW, Dai S (1992). A pseudo-Poisson noise model for simulation of positron emission tomographic projection data. Med Phys.

[35] Santangelo B, Dunn J, Beck K, et al. Modelling continuous arterial blood data from MR-compatible sampler in simultenous pet-MRI experiments. IEEE; 2019 (pp. 750–753)

[36] Tjerkaski J, Cervenka S, Farde L (2020). Kinfitr—an open source tool for reproducible PET modelling: validation and evaluation of test-retest reliability. bioRxiv.

[37] Tonietto M, Rizzo G, Veronese M, et al. Modelling arterial input functions in positron emission tomography dynamic studies. In: Proceedings of the annual international conference of the IEEE engineering in medicine and biology society, EMBS 2015-Novem(August) (pp 2247–2250). 10.1109/EMBC.2015.7318839 10.1109/EMBC.2015.7318839 26736739

[38] Turner H, Firth D. Generalized nonlinear models in R: An overview of the gnm package (2022). https://cran.r-project.org/package=gnm, R package version 1.1-2

[39] van der Weijden CWJ, Mossel P, Bartels AL (2023). Non-invasive kinetic modelling approaches for quantitative analysis of brain PET studies. Eur J Nucl Med Mol Imaging.

[40] Wang X, Feng D. A study on physiological parameter estimation accuracy for tracer kinetic modeling with positron emission tomography (pet). In: 1992 American control conference; 1992 (pp. 1632–1633). 10.23919/ACC.1992.4792385

[41] Wilk MB, Gnanadesikan R (1968). Probability plotting methods for the analysis of data. Biometrika.

[42] Wong KP, Huang SC, Fulham MJ. Evaluation of an input function model that incorporates the injection schedule in FDG-PET studies. In: 2006 IEEE nuclear science symposium conference record. IEEE, San Diego, CA, USA; 2006 (pp. 2086–2090). 10.1109/NSSMIC.2006.354325, http://ieeexplore.ieee.org/document/4179439/

[43] Wood SN (2003). Thin plate regression splines: thin plate regression splines. J R Stat Soc Ser B (Stat Methodol).

[44] Wood SN (2011). Fast stable restricted maximum likelihood and marginal likelihood estimation of semiparametric generalized linear models. J R Stat Soc (B).

[45] Wood SN (2017). Generalized additive models: an introduction with R.

