# Supplementary material for: Parametric and non-parametric Poisson regression for modelling of the arterial input function in positron emission tomography
Source: EJNMMI Phys. 2023 Nov 21;10:72. doi: 10.1186/s40658-023-00591-2 (PMC10663416; doi:10.1186/s40658-023-00591-2)
Supplement: Supplementary file 1 — Additional file 1. Supplementary Materials. [file 40658_2023_591_MOESM1_ESM.pdf]

Parametric and Non-parametric Poisson Regression for Modelling of the Arterial Input Function in Positron Emission Tomography

Granville J. Matheson[1,2,3,4*], Liner Ge[1], Mengyu Zhang[1,5], Bingyu Sun[1,6], Yuqi Tu[1], Francesca Zanderigo[2,3], Anton Forsberg Morèn[4], R. Todd Ogden[1,2]

[1]Department of Biostatistics, Columbia University Mailman School of Public Health, New York, 10032 NY, USA.
[2]Department of Psychiatry, Columbia University, New York, 10032 NY, USA.
[3]Molecular Imaging and Neuropathology Division, New York State Psychiatric Institute, 10032 NY, USA.
[4]Department of Clinical Neuroscience, Center for Psychiatry Research, Karolinska Institutet and Stockholm County Council, Stockholm, SE-171 76, Sweden.
[5]Department of Biostatistics and Data Science, School of Public Health, University of Texas Health Science Center at Houston, Houston, 77030 TX, USA.
[6]Department of Neuroscience, University of Rochester Medical Center, Rochester, 14642 NY, USA.

*Corresponding author(s). E-mail(s): granville.matheson@nyspi.columbia.edu;

Supplementary Materials

Supplementary Materials S1: Parametric Nonlinear Poisson Regression

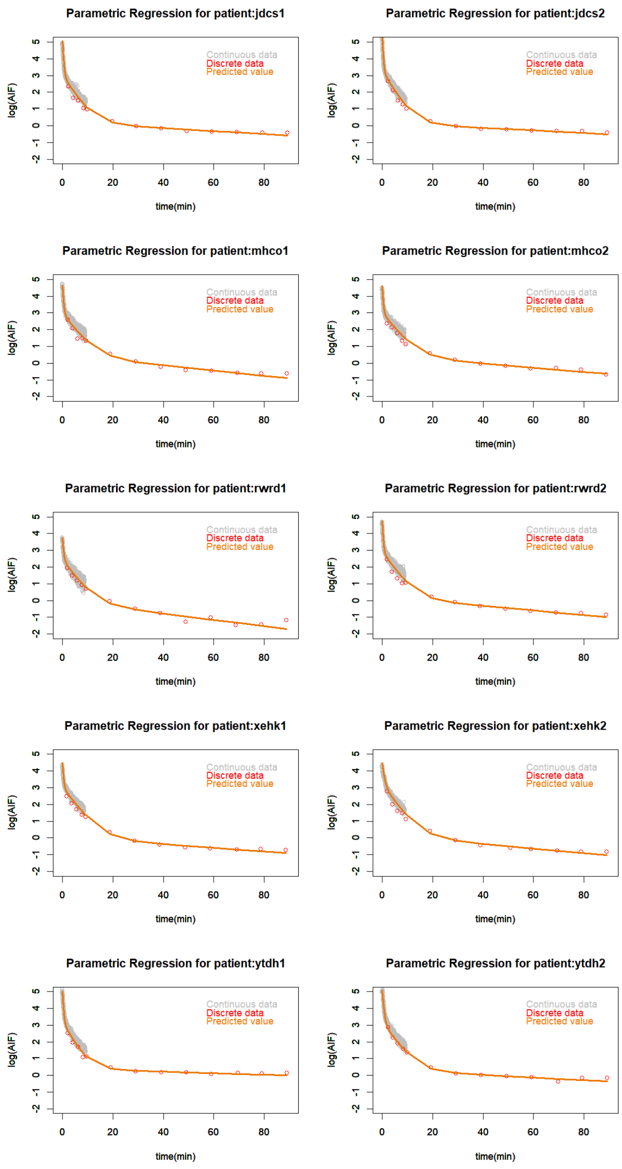

Fig. S1 Predicted values for the parametric tri-exponential model for all examinations

Supplementary Materials S2: Shape-constrained non-parametric Poisson Regression Model

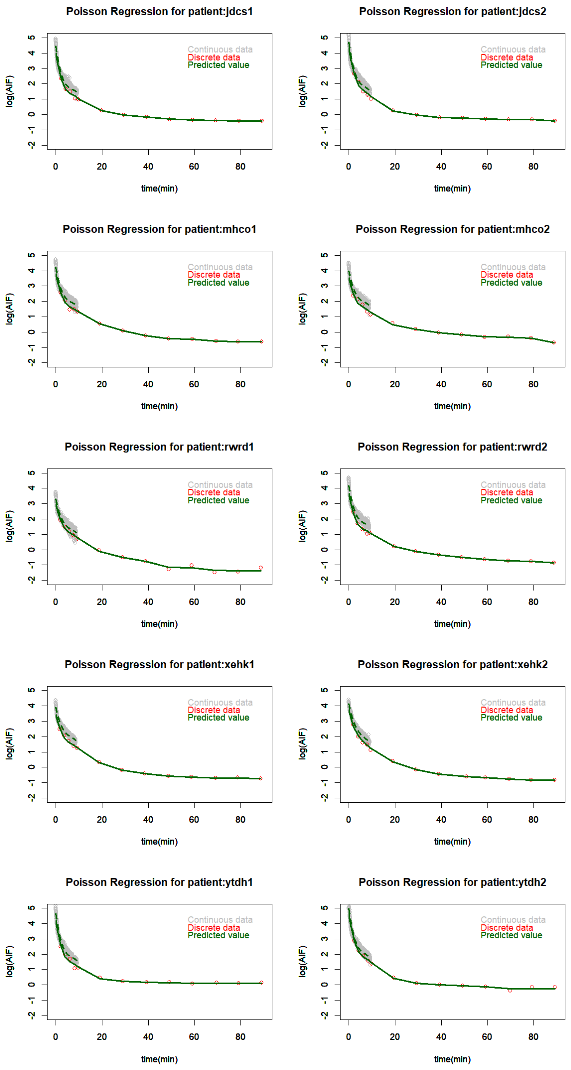

Fig. S2 Predicted values for the shape-constrained non-parametric Poisson model for all examinations

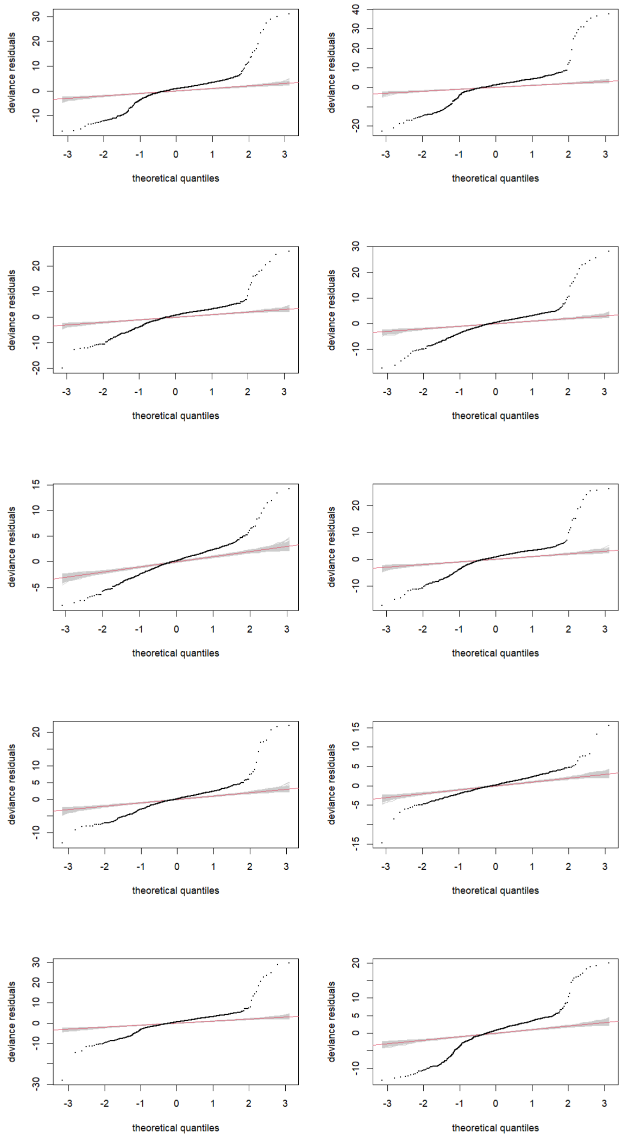

Fig. S3 Residual QQ plots for the shape-constrained non-parametric Poisson model for all examinations

Supplementary Materials S3: Non-parametric Negative Binomial Regression Model

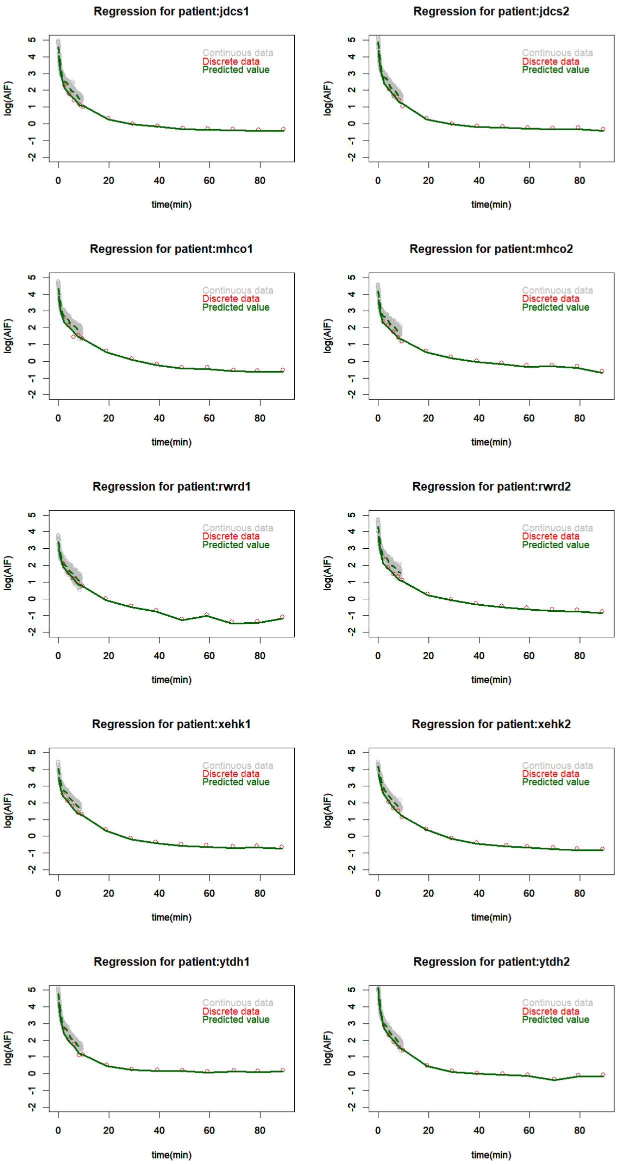

Fig. S4 Predicted values for the non-parametric negative binomial model for all examinations

Supplementary Materials S4: Non-parametric Negative Binomial Regression Model with log-transformed time

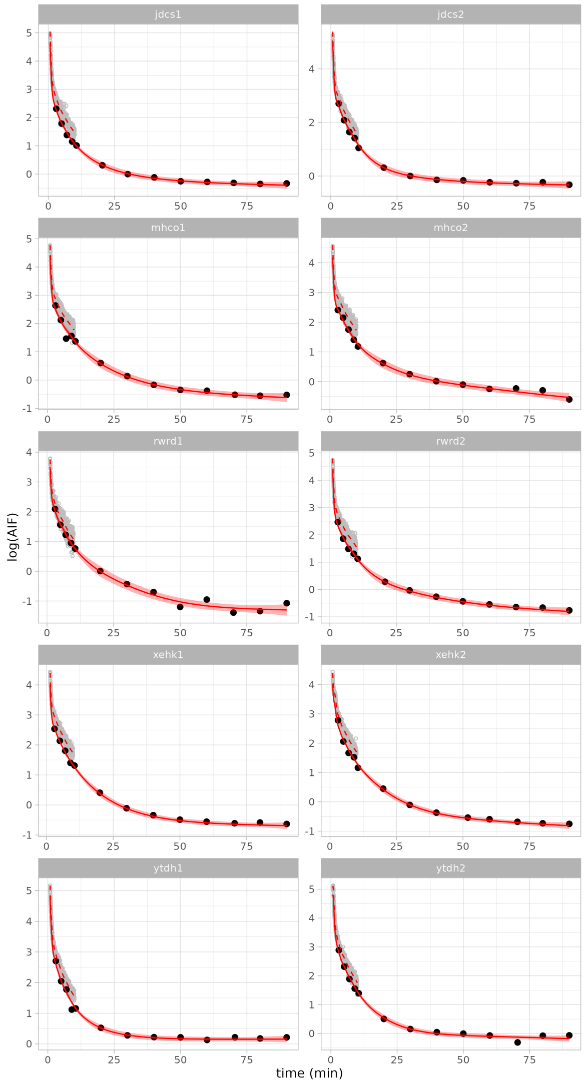

Fig. S5 Predicted values for the non-parametric negative binomial model with log-transformed time for all examinations

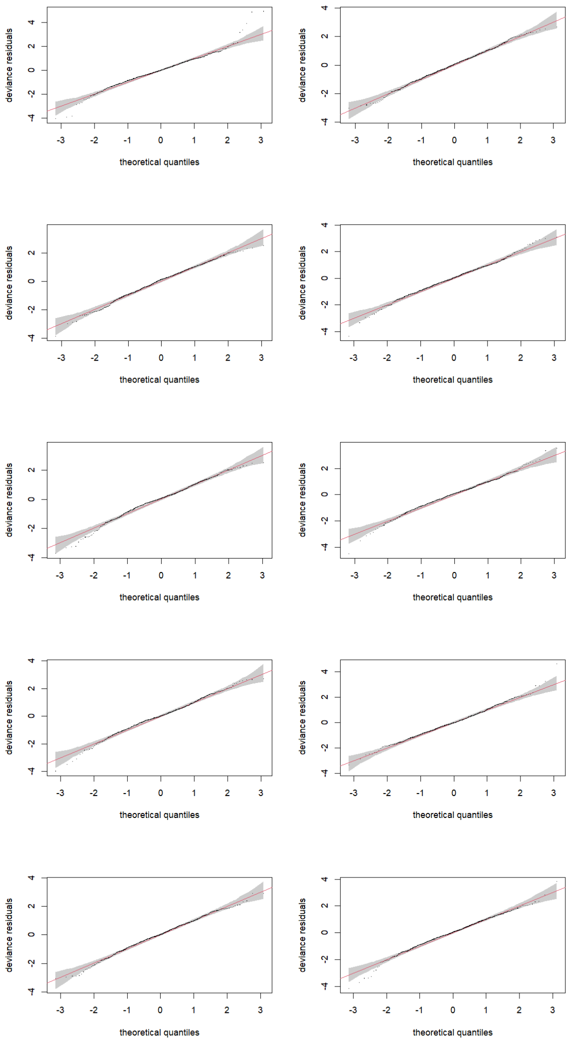

Fig. S6 Residual QQ plots for the non-parametric negative binomial model with log-transformed time for all examinations

Supplementary Materials S5: Hierarchical Negative Binomial Regression Model with log-transformed time

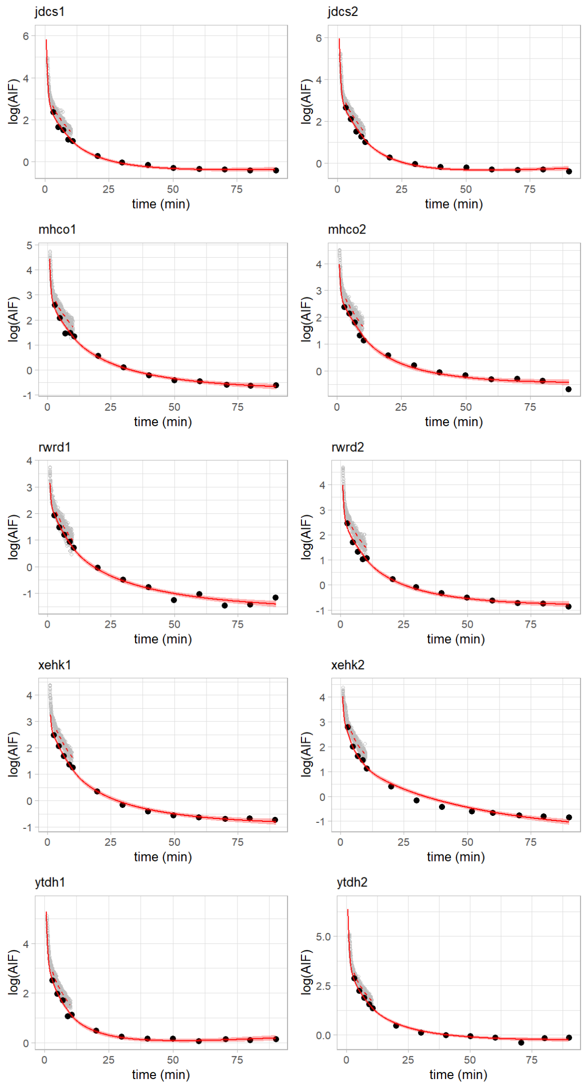

Fig. S7 Predicted values for the hierchical non-parametric negative binomial model with log-transformed time for all examinations

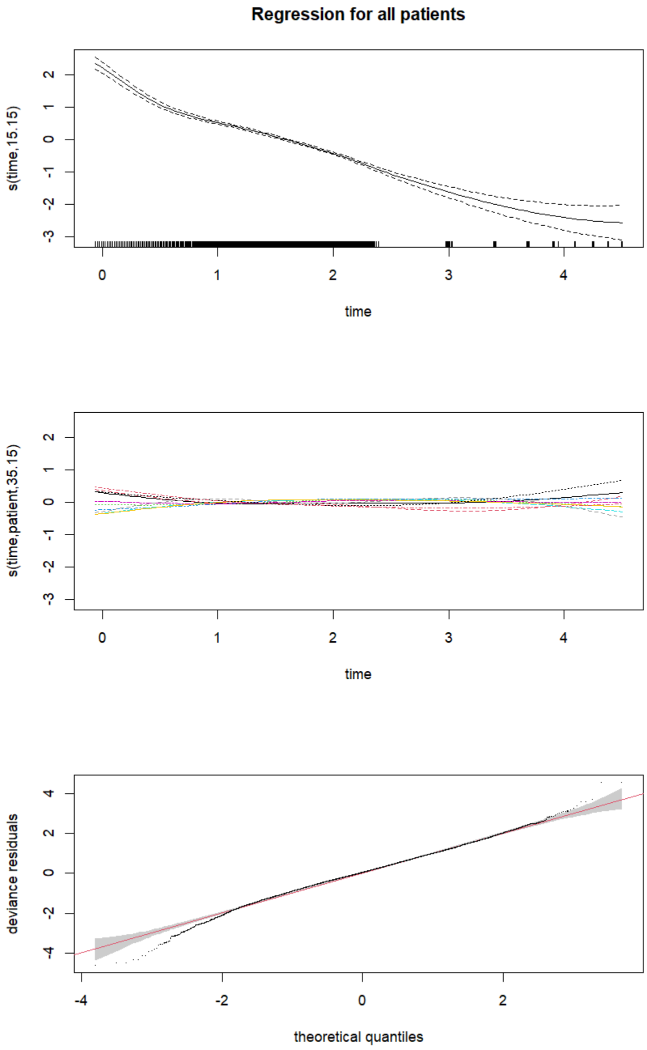

Fig. S8 Partial effect plots and QQ plot for the hierarchical non-parametric negative binomial model with log-transformed time

Supplementary Materials S6: Hierarchical Negative Binomial Regression Model with log-transformed time and missing values

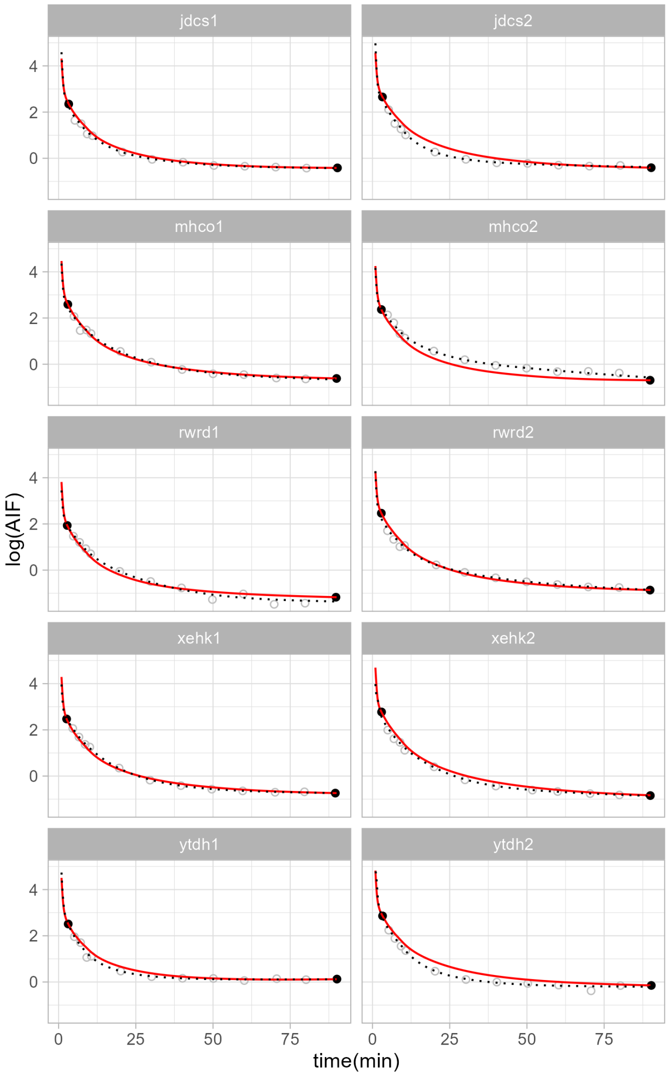

Fig. S9 Fitted curves when only the first and last measurement are available for the specified examination

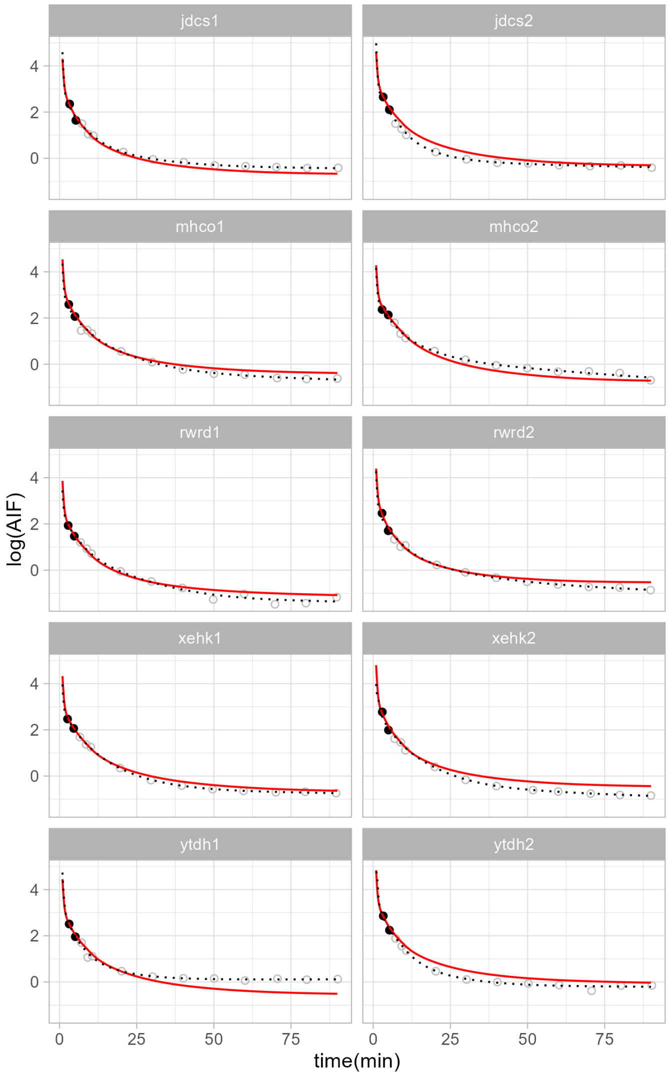

Fig. S10 Fitted curves when only the first two measurement are available for the specified examination

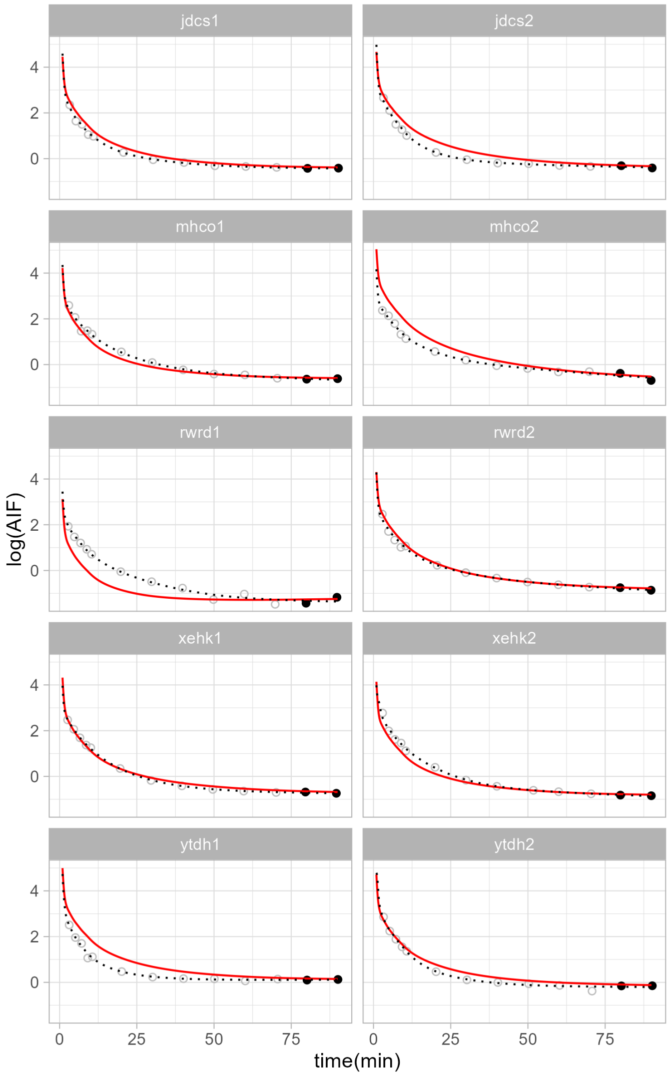

Fig. S11 Fitted curves when only the last two measurement are available for the specified examination

Supplementary Materials S7: Differences between Manual and Automatic Samples with log-transformed time

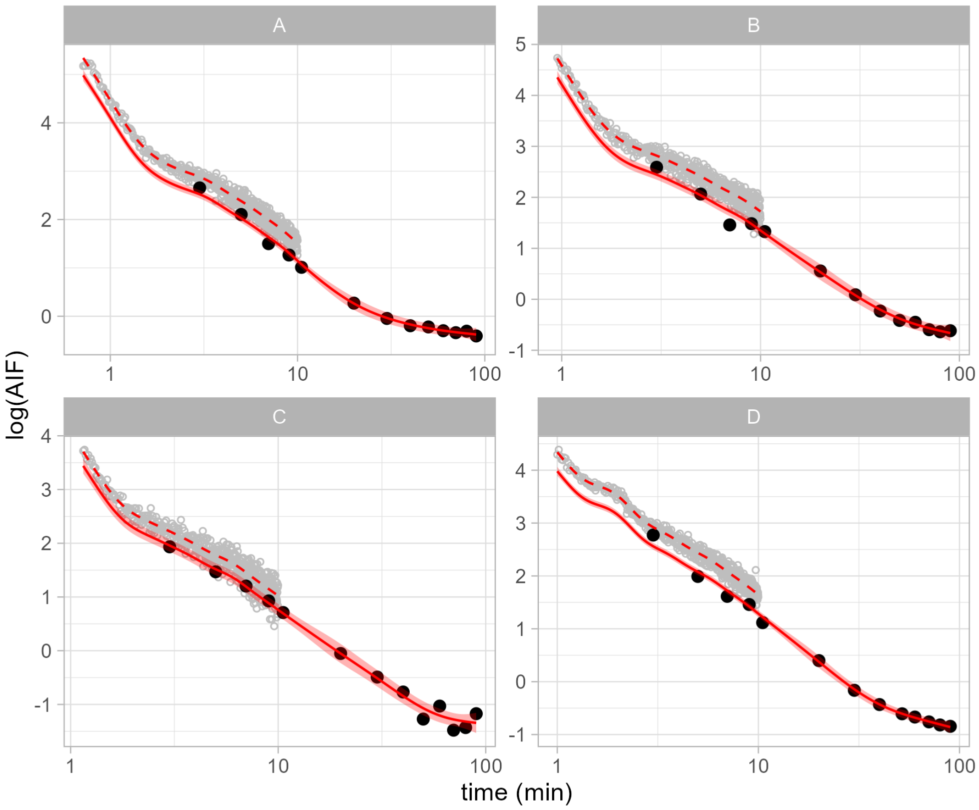

Fig. S12 This figure replicates Figure 1, but with a log-scaling of the x-axis. This demonstrates the proportional differences between the automatic and manual samples appears to have been reasonable consistent over the course of the measurement.
